# Functional Loss of Terminal Complement Complex Protects Rabbits from Injury-Induced Osteoarthritis on Structural and Cellular Level

**DOI:** 10.3390/biom13020216

**Published:** 2023-01-22

**Authors:** Jana Riegger, Helga Joos, Valentin Möhler, Frank Leucht, Katrin Rading, Christian Kubisch, Anita Ignatius, Markus Huber-Lang, Rolf E. Brenner

**Affiliations:** 1 Division for Biochemistry of Joint and Connective Tissue Diseases, Department of Orthopedics, University of Ulm, 89081 Ulm, Germany; 2 Department of Orthopedics, University of Ulm, 89081 Ulm, Germany; 3 Institute of Human Genetics, University Medical Center Hamburg-Eppendorf, 20246 Hamburg, Germany; 4 Institute of Orthopaedic Research and Biomechanics, Ulm University Medical Center, 89081 Ulm, Germany; 5 Institute of Clinical and Experimental Trauma-Immunology, University Hospital Ulm, 89081 Ulm, Germany

**Keywords:** osteoarthritis, terminal complement complex, TCC, ACLT, PTOA, C6, complement activation

## Abstract

The terminal complement complex (TCC) has been described as a potential driver in the pathogenesis of posttraumatic osteoarthritis (PTOA). However, sublytic TCC deposition might also play a crucial role in bone development and regeneration. Therefore, we elucidated the effects of TCC on joint-related tissues using a rabbit PTOA model. In brief, a C6-deficient rabbit breed was characterized on genetic, protein, and functional levels. Anterior cruciate ligament transection (ACLT) was performed in C6-deficient (C6^−/−^) and C6-sufficient (C6^+/−^) rabbits. After eight weeks, the progression of PTOA was determined histologically. Moreover, the structure of the subchondral bone was evaluated by µCT analysis. C6 deficiency could be attributed to a homozygous 3.6 kb deletion within the C6 gene and subsequent loss of the C5b binding site. Serum from C6^−/−^ animals revealed no hemolytic activity. After ACLT surgery, joints of C6^−/−^ rabbits exhibited significantly lower OA scores, including reduced cartilage damage, hypocellularity, cluster formation, and osteophyte number, as well as lower chondrocyte apoptosis rates and synovial prostaglandin E2 levels. Moreover, ACLT surgery significantly decreased the trabecular number in the subchondral bone of C6^−/−^ rabbits. Overall, the absence of TCC protected from injury-induced OA progression but had minor effects on the micro-structure of the subchondral bone.

## 1. Introduction

Complement, as the fluid phase innate immune system, not only plays an important role in infectious and chronic inflammatory diseases but also in trauma situations [1]. Its activation mainly occurs through the classical, alternative and lectin pathways that all merge in the formation of the C5 convertase. This enzyme leads to the cleavage of C5 and thus the generation of anaphylatoxin C5a as well as C5b, initiating the sequential assembly of C6, C7, C8, and C9 to the C5b-9 terminal complement complex (TCC) [2]. Previous studies have demonstrated the potential involvement of TCC during the pathogenesis of degenerative diseases, including Alzheimer`s disease, intervertebral disc degeneration, and osteoarthritis (OA) [3,4,5]. Besides age, obesity, and sex, joint injuries are considered a major risk factor in the development of OA. Struglics et al. reported significantly higher synovial concentrations of complement activation products, including soluble TCC (sTCC), after a knee injury as compared to healthy controls [6]. Moreover, synovial TCC levels were found to be increased in both early- and late-stage OA patients, independent of the etiology of the disease [3]. An important role of TCC in the progression of injury-induced OA was proposed in C6-deficent mice, which were protected to a large extent from cartilage degeneration after destabilization of the medial meniscus [3]. In line, we previously observed that sublytic TCC-deposition contributes to regulated cell death and phenotypical alteration of chondrocytes after cartilage trauma, using a human ex vivo model [4]. 

Although these findings suggest that pharmaceutical complement inhibitors or C6 deficiency and a subsequent lack of TCC formation might prevent or attenuate the development of OA after joint injuries, recent studies indicated that TCC might also contribute to tissue regeneration. In fact, sublytic TCC deposition has been found to promote the survival and proliferation of oligodendrocytes and might play a role in physiological processes, including tissue development and regeneration [7,8,9]. Accordingly, Mödinger et al. revealed that the inhibition of TCC formation led to reduced bone mass and impaired femur fracture healing in C6-deficient mice, implying that the TCC might be crucial for bone biology and potentially involved in bone regeneration [10]. Here, we aimed to characterize the behavior and fate of chondrocytes after anterior cruciate ligament transection (ACLT) in the absence of TCC in more detail.

Previous studies using C6-deficient mice have two shortcomings: first, cellular changes, e.g., cluster formation or hypocellularity, are hardly accessible in mice, due to the thin cartilage and severe cartilage destruction in PTOA models [3]. Second, the serum of the C6-deficient mice exhibited a low level of hemolytic activity, implying residual TCC formation [10]. Therefore, we used a C6-deficient rabbit breed, in which the activation of the complement cascade does not result in TCC formation [11]. Moreover, rabbit cartilage is thicker and closer to human cartilage in regards to cell distribution and structure than that of mice. 

We first describe the underlying mutation causing the C6 deficiency in this animal model, which is important to compare the different models used to study the (patho-) physiological role of C6 deficiency and TCC formation, respectively, and describe some functional consequences in an injury-based OA model. As OA affects the whole joint including the subchondral area, we further wondered whether and to what extent the inability of TCC formation might influence the remodeling of the joint-adjacent bone during OA progression.

## 2. Materials and Methods

### 2.1. Animal Model and Genetic Characterization

A breed of C6-deficient rabbits was established using the last available male animal (kindly provided by Prof. Bhakdi from the Institute of Medical Microbiology and Hygiene, University of Mainz, Germany) and female wildtype New Zealand White rabbits (Crl:KBL(NZW)). For genetic characterization, an Oryctolagus cuniculus chromosome 11 shotgun sequence (NC_013679; position 62,009,331-62,071,633) was used to design intronic PCR primers to amplify the 17 coding exons of the C6 gene [sequences available on request]. DNA from C6-deficient and control animals was isolated by standard procedures. Direct Sanger sequencing of PCR products using Big Dye terminator version 3.1 chemistry was performed on an ABI 3700 DNA Analyzer (Applied Biosystems, Darmstadt, Germany). Primer sequences are provided in the supplements (Table S1).

### 2.2. CH50 Assay and TCC Staining on Erythrocytes

The hemolytic activity of the rabbit serum was evaluated by means of a CH50 assay. In brief, 62.5 μL of a 2%-solution of sheep erythrocytes (ThermoFischer, Waltham, USA) in 20 mM triethanolamine buffer solution (TBS), sensitized with hemolysin (Sigma-Aldrich, Taufkirchen, Germany), and 125 μL of diluted rabbit serum were shaken for 1 h at 37 °C and then diluted with 125 μL TBS on ice. After centrifugation (700 g, 5 min, 4 °C), the optical density of the supernatant was determined at 541 nm and relativized to a 100% lysis control. 

To determine TCC on erythrocytes, the suspension was smeared on a glass slide before centrifugation and stained immunocytologically by means of an anti-rabbit-TCC antibody (clone 42, provided by Sucharit Bhakdi, Mainz, Germany) and an LSAB kit (DAKO, Hamburg, Germany). 

### 2.3. Western Blot Analysis

To assess C6 on a protein level, rabbit serum was separated by SDS-PAGE (Mini-Protean- System, Biorad). The gel (12%) was directly stained with Coomassie (loading control) or used for blotting. After electroblotting onto a nitrocellulose membrane and blocking in 5% BSA (1 h, RT), membranes were incubated with a C6-specific monoclonal antibody (WU6-4; 1:200, Hycultec, Beutelsbach, Germany) overnight at 4 °C. C6 was visualized using an ECL Plex goat-α-mouse IgG-Cy5 (1:1000, Amersham, GE Healthcare, Freiburg, Germany).

### 2.4. ACLT Model

The animal experiment was approved by the local legal authorities (Reg. Number 1156). The genotype of the rabbits was determined by PCR and the presence or absence of functional C6 was confirmed by a CH50 assay in each animal. Overall, 12 female rabbits (15 ± 1.7 months old) were included in the study, with *n* = 6 of each genotype: C6-deficient (homozygous; C6^−/−^) and C6-sufficient (heterozygous animals of the same breed; C6^−/+^). The weight of the rabbits at the time of surgery was identical in both groups (C6-sufficient: 4.3 ± 0.5 kg, C6-deficient: 4.3 ± 0.3 kg). Anterior cruciate ligament transection (ACLT) was performed in the right knee joint of all rabbits. The ACL was totally resected to avoid a spontaneous reconstruction of the ligament. Anesthesia, surgical incision, and postsurgical pain management were performed as previously reported [12]. Unoperated left joints served as control. Animals of different groups were operated alternatingly. Rabbits were euthanized eight weeks after surgery. 

### 2.5. Quantification of PGE2 in the Synovial Fluid

Synovial fluid was aspirated after intraarticular injection of 1 mL PBS (PAA, Egelsbach, Germany) into the joint cavity and repeated flection of the joint as previously described (Riegger et al., 2019, Joos et al., 2015). The concentration of PGE2 was determined by means of an Enzyme Immunoassay Kit (Biotrend, Cologne, Germany).

### 2.6. μCT Analysis 

μCT analysis of the subchondral bone was performed using a μCT scanning device (Skyscan 1172, Kontich, Belgium) with a resolution of 31 μm. The X-ray tube was set to 100 kV, and an aluminum filter with 0.5-mm thickness was applied. Various parameters of the subchondral bone within the medial femoral condyle as the main loading area, including cortical bone mineral density (cBMD), cortical thickness (cTh), bone volume to total volume (BV/TV), trabecular thickness (Tb.Th), trabecular separation (Tb.Sp), and trabecular number (Tb.N), were determined after calibration with two defined densitometric phantoms (250 mg/cm^3^ and 750 mg/cm^3^ hydroxyapatite bone-equivalent density). The region of interest (ROI) was manually selected as follows: in the case of BV/TV, Tb.Th, Tb.Sp, and Tb.N, a defined area of 2 mm^3^ of the trabecular bone in the middle of the medial condyle was chosen. In the case of cBMD and cTh, subchondral sclerotization was assessed by choosing an area with a width of 1.9 mm and a height of 11 mm. The subchondral cortical plate was defined as ROI with a depth of 1 mm (Figure S1).

### 2.7. Histopathological Assessment and TUNEL Staining

The synovial membrane, femoral condyles, and tibia plateau were fixed in 4% paraformaldehyde. Additionally, the femoral condyles and the tibia plateau were decalcified (20% EDTA) for 4 weeks. Tissues were embedded in paraffin, cut, and dewaxed/ rehydrated prior to staining. The femoral condyles and tibia plateaus were stained with safranin-O (SafO) and fast green (counterstaining), while hematoxylin/eosin (H&E) staining was performed in the case of the synovial membrane. For the OA assessment, a modified synovial and OA score, respectively, was used according to the OARSI scoring system (Tables S2 and S3 in the supplementary material) (Laverty et al., 2010). The scoring was performed independently and blinded by two observers.

The apoptosis rate was determined by means of a commercially available TUNEL assay (Promega, Walldorf, Germany) in accordance with the manufacturer’s instructions. Images were taken from the superficial/ transitional zone as well as from the deeper zone (tidemark) as delineated in the supplementary material (Figure S2).

### 2.8. Statistical Analyses

For statistical analysis, a two-way ANOVA with Sidak multiple comparison test was performed by means of GraphPad Prism version 8.4.3 (GraphPad Software). Data sets with *n* ≥ 5 were tested for outliers using the Grubbs outlier test. Outliers were not included in statistical analyses. The significance level was set to α = 0.05. “n” indicates the number of animals (independent, biological replicates) included in the analysis.

## 3. Results

### 3.1. Inability to Form Functional TCC in C6-Deficient Rabbits Results from the Loss of the C5b Binding Site in C6

**Figure 1:**
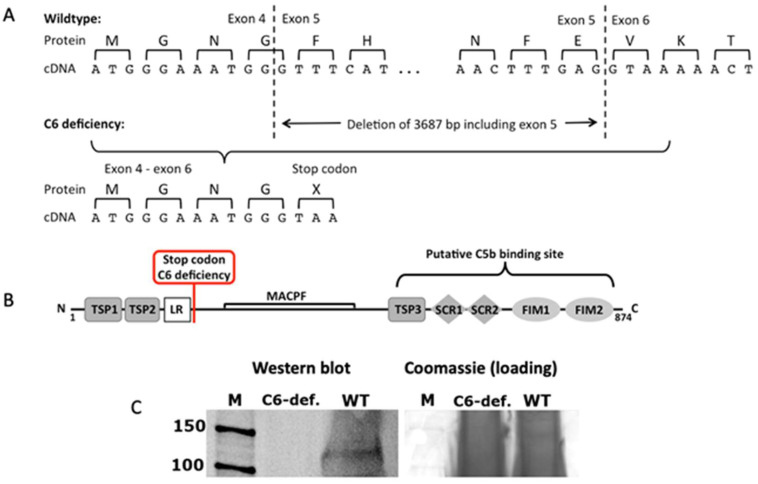
Characterization of the mutated C6 gene. (**A**,**B**) Structure of the rabbit C6 protein according to transcript C6-201, ENSOCUT00000001933 (Ensemble release 84) with indication of the putative C5b binding site 2 and the stop codon caused by the mutation; TSP: thrombospondin type 1 repeat, LR: Low-density lipoprotein receptor class A repeat, MACPF: Membrane attack complex component/perforin domain, SCR: short consensus repeats, FIM: factor I modul. (**C**) Consequence of the genomic deletion in the C6 gene of C6-deficient rabbits on protein level. Serum of C6-deficient (C6-def.) and wildtype rabbits (WT) was resolved by 12% SDS-PAGE and coomassie-stained or used for western blot and detected with a C6-specific antibody; M: protein marker.

Although the lack of C6, which precludes the formation of a functional TCC in the C6-deficient rabbit breed, was first described over 50 years ago [11] and subsequently proved to represent a valuable animal model in complement research, the underlying mutation remained unidentified. Our analyses revealed that all exons–except exon 5–could be amplified by intronic primers and showed no putative mutation after direct sequencing of PCR products. Further long-range PCR amplification with primers in intron 4 and 5 identified a homozygous deletion of 3.673 basepairs (c.588-1207_726+2327del3673) including the complete exon 5. Gene expression analysis of blood samples and direct sequencing of PCR products confirmed the skipping of exon 5 on the cDNA level. This exon skipping is predicted to either lead to nonsense-mediated decay or in the case of the generation of a stable protein to a frameshift with a consecutive premature stop codon at the beginning of exon 6, resulting in the loss of the C5b binding site (Figure 1A,B). This binding site has previously been located at the third thrombospondin repeat of C6 [13]. Subsequent western blot analyses failed to detect C6 in the serum of C6-deficient animals (Figure 1C). Additional analyses, using a polyclonal C6-specific antibody (LSBio, Eching, Germany, data not shown) confirmed that neither the whole protein nor smaller products could be detected. However, it remained unclear if the truncated mRNA is translated.

**Figure 2:**
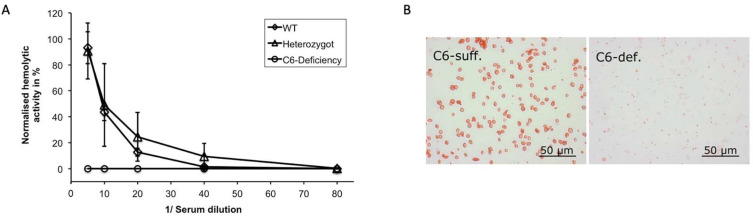
Functional testing of TCC in rabbit serum. (**A**) Hemolytic activity of rabbit serum derived from WT (*n* = 3), heterozygote (*n* = 8) and C6-deficient (*n* = 9) animals was determined by means of a CH50 assay; curves represent column means at different dilutions with standard deviation. (**B**) Anti-TCC immunocytochemical staining of sheep erythrocytes exposed to C6-sufficient (C6-suff.) and C6-deficient (C6-def.) serum, respectively.

By means of a CH50 assay, we confirmed the inability of C6-deficient animals to form functional TCC due to the mutation in C6. As expected, serum derived from homozygous animals lacked the ability to lyse sensitized erythrocytes, while serum of heterozygous rabbits exhibited no impairment in hemolysis (Figure 2A). Interestingly, the hemolytic activity of C6^+/−^-derived serum was slightly higher as compared to that of wildtype (WT) animals as observed in the dilution curve. In contrast to erythrocytes exposed to C6^+/−^ serum, no TCC deposition could be detected on the erythrocyte membranes after incubation with the serum of C6^−/−^ rabbits (Figure 2B).

### 3.2. C6-Deficiency Attenuates Structural Cartilage Damage and Cluster Formation after ACLT Surgery

**Figure 3:**
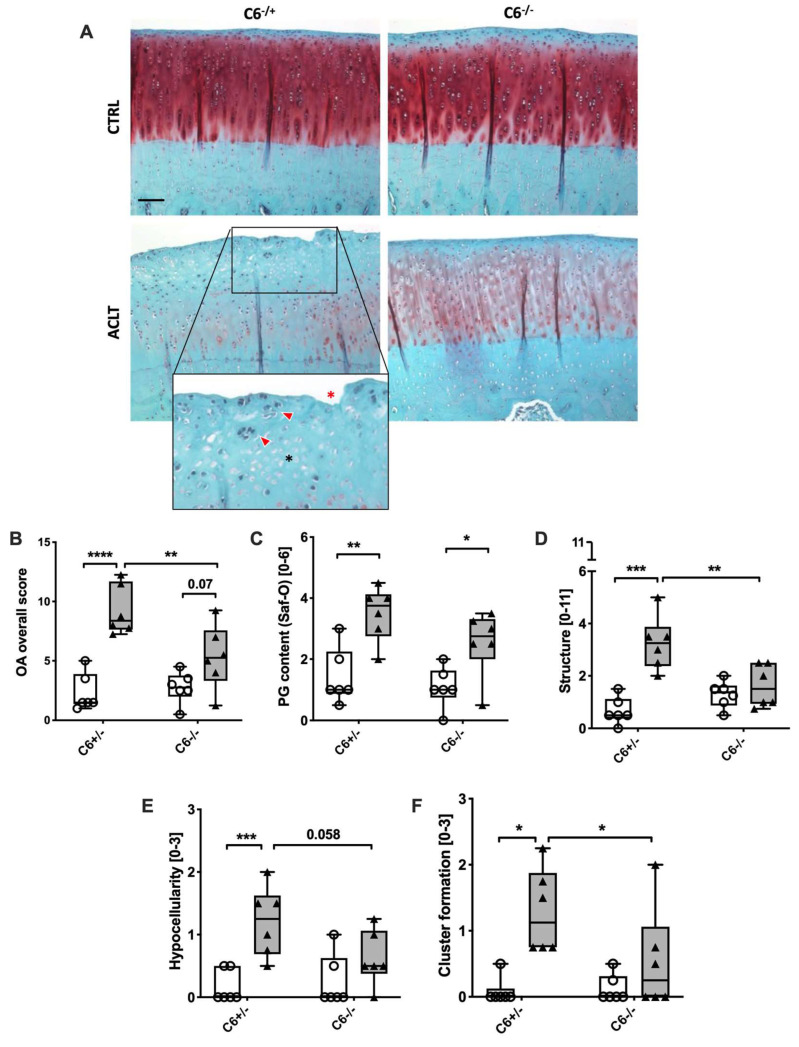
Histopathologic assessment of medial condyle sections. (**A**) Exemplary images of SafO-stained medial condyles of both experimental groups. Cell clusters are indicated by red arrowheads; black asterisk indicates hypocellular region; red asterisk indicates irregular surface; the black bar represents 50 µm. (**B–E**) Corresponding statistical analysis of the overall score and single parameters of the histopathological assessment: (**B**) overall score, (**C**) proteoglycan content/ SafO staining intensities, (**D**) structure/ surface integrity, (**E**) hypocellularity, and (**F**) cluster formation. Data are charted as box plots with median and whiskers min to max; white boxes= control joint, grey boxes = ACLT-operated joints. Statistically significant differences between groups (*n* = 6 each) were depicted as: *: *p* ≤ 0.05, **: *p* ≤ 0.01, and ***: *p* ≤ 0.001, ****: *p* ≤ 0.0001.

Whether ACLT-related OA in rabbits is more pronounced in the medial or lateral condyle still remains controversially discussed [14,15,16]. In our study, we performed a histopathological assessment of both the medial and lateral femoral condyles after safranin-O staining to evaluate osteoarthritic changes eight weeks after ACLT surgery. ACLT-induced cartilage degeneration was more pronounced on the medial side (Figure 3A,B) as compared to the lateral condyle, which exhibited only mild changes (Figure S3). Moreover, no alterations were found in the tibia plateau (Figure S5).

While ACLT surgery significantly increased the overall OA score in C6-sufficient animals as compared to the unoperated contralateral side (2-fold; *p* = 0.0002), the manifestation of OA-associated changes was significantly lower in the case of the C6-deficient group (*p* = 0.006; Figure 3B). This difference was largely reflected in the single OA criteria, except PG content (Figure 3C). Indeed, ACLT-related changes in C6-deficient rabbits were significantly lower in the case of cartilage structure/ damage (2-fold; *p* = 0.007; Figure 3D), hypocellularity (2-fold; *p* = 0.058; Figure 3E), and cluster formation (2.4-fold; *p* = 0.04; Figure 3F) as compared to the C6-sufficient group. 

**Figure 4:**
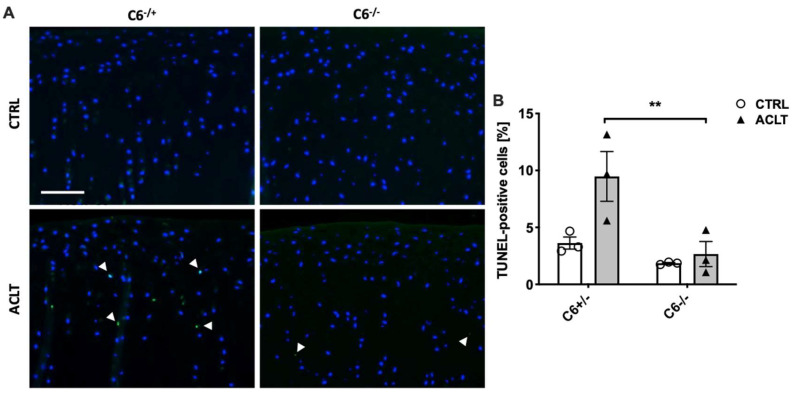
TUNEL staining of medial condyle sections. (**A**) Exemplary images of TUNEL-stained cartilage. Images were taken from the superficial and transitional zone. TUNEL-positive cells (green) are exemplarily indicated by white arrowheads; the white bar represents 100 µm. (**B**) Corresponding statistical analysis of the apoptosis rate (percentage of TUNEL-positive chondrocytes). Data are charted as scattered bars with mean and SEM; white boxes = control joint, grey boxes = ACLT-operated joints. Statistically significant differences between groups (*n* = 3 each) were depicted as: **: *p* ≤ 0.01.

To determine whether increased hypocellularity in C6-sufficient animals was caused by TCC-induced apoptosis as previously described [4], we performed a TUNEL staining. As expected, we found a significantly higher percentage of TUNEL-positive chondrocytes in the superficial and transitional zone of the cartilage in ACLT-operated C6-sufficient rabbits as compared to the C6-deficient group (2.3-fold; *p* = 0.009; Figure 4A,B). The number of TUNEL-positive chondrocytes in the cartilage of C6-sufficient rabbits after ACLT surgery was also enhanced in deeper zones (tidemark) as shown in the supplementary material (Figure S3).

### 3.3. C6-Deficiency Reduces Osteophyte Formation but Has No Significant Effect on Synovial Inflammation after ACLT Surgery

**Figure 5:**
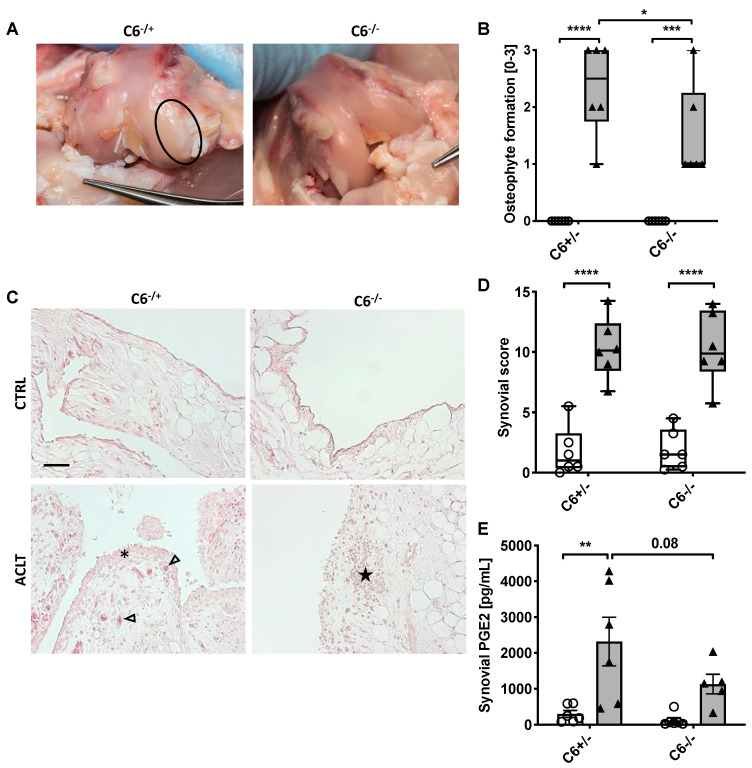
Evaluation of osteophyte formation and synovial inflammation. (**A**) Exemplary images of ACLT-operated joints after 8 weeks; macroscopic femoral osteophytes are indicated by a black circle. (**B**) Corresponding statistical analysis of macroscopically detectable osteophytes. (**C**) Exemplary images of Hematoxylin/ Eosin-stained synovial membrane of both experimental groups. Endothelial cells are indicated by black arrowheads; black asterisk indicates proliferation and hypertrophy of synoviocytes in lining layer; black star indicates lymphocyte aggregation; the black bar represents 50 µm. (**D**) Statistical analysis of the synovial score and (**E**) determination of synovial PGE2 concentrations. Data are charted as (**B**,**D**) box plots with median and whiskers min to max or (**E**) scattered bars with mean and SEM; white boxes/ bars= control joint, grey boxes/ bars= ACLT-operated joints. Statistically significant differences between groups (*n* = 6 each) were depicted as: *: *p* ≤ 0.05, **: *p* ≤ 0.01, ***: *p* ≤ 0.001, and ****: *p* ≤ 0.0001. Pictures in (A) were modified from [20].

Osteophyte formation is an intrinsic response toward joint destabilization and was, therefore, evaluated by means of a macroscopic assessment [17,18,19]. As expected, ACLT surgery significantly induced the formation of femoral osteophytes, irrespective of the genotype, whereas unoperated joints presented no pathological findings macroscopically (Figure 5A,B). Interestingly, osteophyte formation was significantly higher in C6-sufficient rabbits as compared to the C6-deficient group ( + 50%; *p* = 0.04). 

Synovial inflammation and the subsequent release of pro-inflammatory mediators play an important role in the pathogenesis of PTOA. Consequently, synovitis was assessed by a histological score (Figure 5C). As compared to the respective unoperated contralateral joint, histological changes of the synovial membrane were found to be significant in both genotypes after ACLT (~5.7-fold, *p* < 0.0001; Figure 5D). The most affected parameters were the proliferation and hypertrophy of synoviocytes, inflammatory infiltration and proliferation of fibroblasts as well as angiogenesis. No infiltration of granulocytes could be detected in any group. Although ACLT-related synovitis was similar in both genotypes, synovial levels of PGE2 were clearly attenuated in synovial fluid derived from C6-deficient rabbits as compared to the C6-sufficient group (-2-fold%, *p* = 0.08) (Figure 5E).

### 3.4. ACLT-Related Alteration of the Subchondral Bone Is More Pronounced in C6-Deficient Rabbits

**Figure 6:**
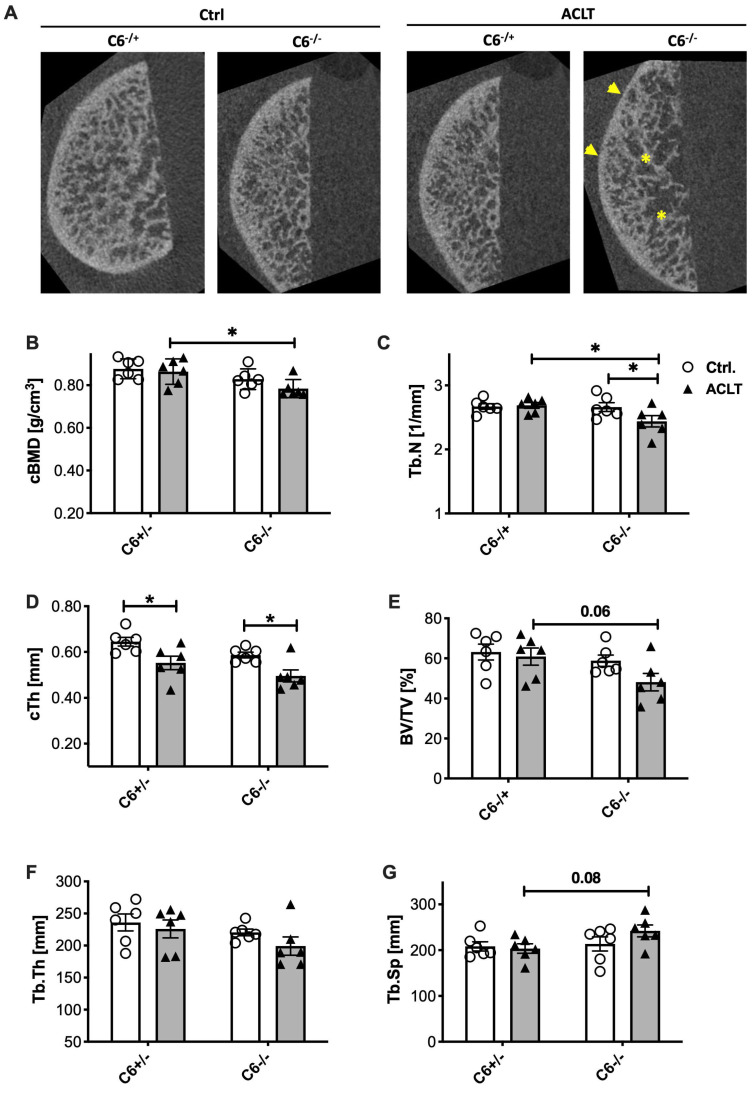
Structural analysis of subchondral bone. (**A**) Exemplary image of µCT analysis after 8 weeks; reduction in cortical thickness is indicated by yellow arrowheads, reduction in BV/TV and Tb.N., respectively, is indicated by yellow asterisks. Statistical analysis of (**B**) cBMD, (**C**) Tb.N, (**D**) cTh, (**E**) BV/TV, (**F**) Tb.Th, and (**G**) Tb.Sp. Data are displayed as scattered bars with mean and SEM; white bars= control joint, grey bars= ACLT-operated joints. Statistically significant differences between groups (*n* = 6 each) were depicted as: *: *p* ≤ 0.05.

Eight weeks after ACLT surgery, changes in the subchondral bone were assessed by means of a µCT analysis (Figure 6A). Overall, C6-deficient animals displayed no significant alterations of the subchondral bone. While no change in cBMD and Tb.N was found in C6-sufficient rabbits after ACLT surgery, both parameters were lowered in the C6-deficient group (cBMD: −12%; *p* = 0.06; Tb.N: −10%; *p* = 0.02) (Figure 6B,C). Moreover, ACLT surgery resulted in a significant reduction in cTh, irrespective of the genotype (Figure 6D). Although ACLT surgery had generally no significant effect on BV/TV, Tb.Th, Tb.Sp in trabecular bone, operated joints of C6-deficient rabbits exhibited a notable reduction in BV/TV (−31%; *p* = 0.06) and Tb.Th (−18%; *p* = 0.276), as well as an increase in the case of Tb.Sp (+16%; *p* = 0.08), as compared to the C6-sufficient counterpart (Figure 6E–G). 

## 4. Discussion

Injury-associated complement activation and subsequent TCC formation have previously been discussed as potential drivers during the pathogenesis of PTOA [3,4]. However, reports deriving from other research fields imply that the TCC may not only act as a pathophysiological mediator but might also regulate physiological and even regenerative processes [7,8,9,10]. Indeed, having a closer look at the different joint-related tissues affected by OA, we here observed for the first time not only protective but also potentially adverse consequences of the lack of TCC in an in vivo model of PTOA. These novel findings provide an important insight into the pathophysiological role of TCC in the context of injury-induced OA.

Previous studies dealing with this unique rabbit breed, which carries a naturally occurring mutation in C6, reported that the animals were protected from synovitis in short-term investigations using urate crystal- and antigen-induced arthritis models [21,22]. In the present study, we chose a surgically-induced OA model, which might be less inflammation-driven than those arthritis models. The ACLT model is based on joint destabilization and subsequent repetitive overloading of the joint, resulting in cartilage degeneration over time. 

Although this C6-deficient rabbit breed has been used for many years [11,23,24], we here first characterized the underlying genetic mutation, demonstrating a deletion that exclusively affects the C6 gene and predicts a truncated mRNA. In accordance with the literature, no intact C6 protein, TCC formation or lytic activity after complement activation could be detected in C6-deficient serum in contrast to the serum of wildtype or heterozygous animals [11]. Therefore, this rabbit model is well suited to study the consequences of a selective total C6 deficiency, which is noteworthy, because low levels of hemolytic activity due to remaining TCC formation were previously observed in C6-deficient mice [10]. In sum, seven point mutations within the C6 gene have been described in this mouse strain, of which four mutations lead to single amino acid substitutions, most likely causing the disruption of the tertiary structure of the protein. While the complete C6 protein is predicted to be translated in C6-deficient mice, structural alterations of the complement component are thought to impair its lytic activity [25]. 

In our rabbit model, the genomic deletion leads to a truncated mRNA that definitely lacks the binding sites for C5b, which can be considered as a substantial difference in comparison to the mouse model [3], exhibiting a certain level of residual hemolytic activity. Moreover, it should be noted that lower C6 and C9 levels have been reported in female animals of certain frequently used mouse strains as compared to male littermates. This limitation in available complement components results in reduced activity of the terminal complement pathway [26]. In contrast, no age- or sex-dependent difference was found in the case of C6 in rabbits [27]. Unimpaired hemolytic activity of C6^+/−^ serum indicated that C6 levels are not rate limiting for TCC formation, thus heterogenous rabbits were considered as C6-sufficient in the present study. Furthermore, adhering to the 3Rs in animal research encouraged us to use heterogeneous rabbits as a suitable control.

Besides potential differences in osteo-regeneration, which are discussed below, our study revealed further indications of altered cellular response due to the lack of TCC. Although cell cluster formation within the cartilage is an accepted criterion used for the histological assessment of OA, it remains controversially discussed whether the clusters result from dedifferentiated and thus dysfunctional chondrocytes or represent a regenerative attempt of proliferating stem progenitor cells [28,29]. Reduced cluster formation in C6-deficient rabbits might thus be interpreted in different ways, either as an indicator of attenuated OA progression or as a result of missing pro-mitotic signals. In fact, sublytic TCC deposition has been identified as a trigger of proliferation in various cell types and with disparate impacts, ranging from tissue degeneration to regeneration [7,30,31]. However, the pro-mitotic effects of TCC on chondrocytes have not been reported so far. In terms of reduced osteophyte formation, we assume that the inability to form functional TCC might result in impaired osteogenic activity and in particular endochondral ossification, as previously described [10]. Moreover, the reduction in osteophyte number and size might be associated with the finding of attenuated PGE2 levels. On one hand, PGE2 has been described as a mediator involved in osteophyte formation via E prostanoid (EP) rhodopsin-like G-protein coupled receptor 4 [32], on the other hand, high levels of PGE2 have been found to be produced by osteophytes under pro-inflammatory conditions [33]. 

Regarding the reduced bone mass in C6-deficient mice [10], we paid special attention to the subchondral bone of the medial femoral condyle. While no major abnormalities were observed in the unoperated control joint, cBMD, BV/TV, Tb.N, as well as Tb.Th were somehow decreased, and Tb.Sp increased in ACLT-operated joint of C6-deficient rabbits as compared to the corresponding C6-sufficient group. Although the effects are rather mild, the µCT analysis implies a slightly higher impact of ACLT on the subchondral bone micro-structure of C6-deficient rabbits. In the literature, similar changes associated with an increased bone turnover have been reported in the distal femur and proximal tibia eight weeks after ACLT surgery in 9- to 12-month-old male New Zealand white rabbits [34,35]. 

The fact that we could not find any changes in the subchondral bone of the C6-sufficient animals might result from differences in the genetic background, age, sex, as well as analyzed region and time point. It is noteworthy, that enhanced bone remodeling and subsequent deterioration of the subchondral bone in OA patients is rather associated with cartilage defects and observed in early OA, while subchondral bone sclerosis is widely classified as a hallmark of end-stage OA [36]. In line with these clinical observations, subchondral bone changes were found to return to normal at 12 weeks in rabbits and already 6 weeks after ACLT surgery in mice [18,37]. Considering the generally low impact of ACLT on subchondral bone remodeling, investigation of bone healing using an osteochondral defect or even fracture model in C6-deficient rabbits might be more meaningful to clarify potentially compromised bone remodeling and tissue regeneration, respectively, due to an impairment of osteoanabolic processes as previously reported in mice [10]. 

Reduced hypocellularity and a lower apoptosis rate in cartilage tissue of C6-deficient rabbits after ACLT further indicate potential cell protection due to the lack of TCC. Our previous studies on TCC-mediated pathomechanisms after cartilage injury clearly demonstrated that even sublytic TCC deposition can trigger chondrocyte cell death, most likely by initiating both caspase-dependent and -independent pathways, resulting in apoptosis and regulated necrosis, respectively [4]. In contrast to our rabbit in vivo cartilage trauma model, in which a singular trauma is directly applied on the cartilage surface by means of a spring-loaded impact device, we observed a lower influence on chondrocyte death and subsequent hypocellularity after ACLT surgery [38]. Enhanced loss of chondrocytes after direct cartilage trauma might be explained by the high focal impact energy, which might be more harmful compared to the repetitive overload in the ACLT model.

In agreement with Wang et al., who described a significant attenuation of DMM-induced OA and synovitis in C6-deficient mice [3], we observed a significant reduction in the overall OA score, as well as a decrease in osteophyte formation, after ACLT surgery in our rabbit model. However, no influence was found regarding ACLT-induced synovial inflammation in C6-deficient rabbits. Additionally, our model allowed the assessment of hypocellularity and cluster formation, which both were lowered in C6-deficient animals. Due to the thin cartilage in mice and subsequent severe destruction during injury-induced OA progression, these parameters have not been addressed before. Without ACLT injury no phenotypic difference in the knee joints could be observed in our rabbits at the age of analysis. Moreover, there was no influence of the C6 genotype on body weight or general postoperative motor activity, arguing against different biomechanical loading. Our findings emphasize that the TCC might play an important role in the pathogenesis of OA but it is not the only mediator involved in this rather complex process. It is well known that supraphysiological loading, as caused by ACLT surgery, leads to the activation of mechano-sensitive pathways and oxidative stress in chondrocytes, consequently inducing regulated cell death and catabolic enzyme expression [29,39,40]. Due to the repetitive compression of the cartilage and subsequent cell death, both matrix-related and intracellular damage associated molecular pattern (DAMPs) are released into the synovial fluid. These DAMPs can bind to pattern recognition receptors on different cell types, including synovial fibroblasts and macrophages, causing proliferation and enhanced expression of pro-inflammatory cytokines [41]. Moreover, the anaphylatoxin generation of C3a and C5a is not affected by C6 deficiency and is well-documented to play a crucial role in synovial inflammation [42,43]. 

## 5. Conclusions

In summary, the detailed genetic characterization of our rabbit model offers a unique possibility to address related questions with a focus on the role of TCC in OA progression in a non-rodent model. Moreover, the usage of a rabbit model allowed for a more detailed analysis of cellular alteration in response to ACLT, revealing a decrease in cartilage cell clusters and hypocellularity (chondrocyte apoptosis) in C6-deficient animals. Overall, we provide the first evidence that C6-deficient rabbits are partially protected from OA development after ACLT surgery, but might also have minor disadvantages regarding subchondral bone remodeling.

## Data Availability

Not applicable.

## Supplementary Materials

The following supporting information can be downloaded at: https://www.mdpi.com/article/10.3390/biom13020216/s1, Figure S1: Assessment of the condylar bone by µCT; Figure S2: Zones considered in the TUNEL assay; Figure S3: Histopathologic assessment of lateral condyle sections; Figure S4: Histopathologic assessment of tibia plateau sections; Figure S5: TUNEL staining; Table S1: Primer for genetic characterization of the C6-deficiency; Table S2: OA scoring system; Table S3: Synovitis scoring system.
