# Supplementary material for: Functional Loss of Terminal Complement Complex Protects Rabbits from Injury-Induced Osteoarthritis on Structural and Cellular Level
Source: Biomolecules. 2023 Jan 22;13(2):216. doi: 10.3390/biom13020216 (PMC9953363; doi:10.3390/biom13020216)
Supplement: Supplementary file 1 [file biomolecules-13-00216-s001.zip › biomolecules-2166809-supplementary.pdf]

Supplementary Material

Supplementary Tables

Table S1: **Primer for genetic characterization of the C6-deficiency.**

Forward primer in intron 4 and reverse primer in intron 5 for amplification of a junction fragment.	For: 5´-attaaagccatgccgataac-3´<br>Rev: 5´-tgaatttaacagttggaggatc-3´
Primer for blood cDNA analyses	For: 5´-aatgactgtggagacaattcag-3´<br>Rev: 5´-agaaatccgtttccaagtcatc-3´

Table S2: OA scoring system. Modified score for histochemical/histological assessment of articular cartilage changes in OA according to Laverty et al. 2010, Osteoarthritis and Cartilage 18 (Suppl 3), pp53–65 and Riegger et al. 2019, International Journal of Molecular Sciences 20(12), 2916.

Safranin-O- Fast green staining of weight bearing region	
0	Uniform staining throughout articular cartilage
1	Loss of staining in 1/3 superficial zone of hyaline cartilage
2	Loss of staining in 1/3 superficial and lower zone of hyaline cartilage
3	Additionally, slight reduction in central zone of hyaline cartilage
4	Overall, poor proteoglycan staining
5	Only some cells/ cluster stained posititive
6	No staining
Structure	
0	Normal
1	Surface irregularities
2	Fissures in <50% surface
3	Fissures in ≥50% surface
4	Erosion 1/3 hyaline cartilage <50% surface
5	Erosion 1/3 hyaline cartilage ≥50% surface
6	Erosion 2/3 hyaline cartilage <50% surface
7	Erosion 2/3 hyaline cartilage ≥50% surface
8	Full depth erosion hyaline cartilage <50% surface
9	Full depth erosion hyaline cartilage ≥50% surface
10	Full depth erosion hyaline and calcified cartilage to the subchondral bone <50% surface
11	Full depth erosion hyaline and calcified cartilage to the subchondral bone ≥50% surface

Hypocellularity	
0	Normal
1	Focal decrease in cells
2	Multifocal decrease in cells
3	Confluent decrease in cells
4	Largely de-cellularized
Cluster formation	
0	Normal
1	< 4 Clusters
2	≥ 4 Clusters
3	≥ 8 Clusters

Table S3: Synovitis scoring system. Modified Score for histopathological assessment of OA synoviopathy according to Laverty et al. 2010, Osteoarthritis and Cartilage 18, Suppl 3, pp53–65.

Synoviocytes		
Proliferation	0: Normal	One layer
	1: Slight	Up to two cells deep
	2: Moderate	Up to four cells deep
	3: Severe	Up to more than four cells deep
Hypertrophy	0: Normal	Squamous
	1: Slight	Cuboidal
	2: Moderate	Cylindrical
	3: Severe	Cylindrical with cytoplasmic protrusions
Inflammatory infiltrate		
Granulocytic and lymphoplasmacytic infiltrate	0: Absent	
	1: Slight	Few cells
	2: Moderate	Some foci of cells
	3: Severe	Diffuse infiltration
Lymphoplasmacytic aggregates/follicles	0: Absent	
	1: Single	1
	2: Many	>1
Synovial stroma		
Villous hyperplasia	0: Absent	No villi/smooth waves
	1: Slight	Some small villi
	2: Moderate	Multifocal villous hyperplasia
	3: Severe	Diffuse villous hyperplasia

Proliferation of fibroblasts/fibrocytes	0: Absent<br>1: Slight<br>2: Moderate<br>3: Severe	Normal cellular density<br>Mild proliferation<br>Moderate proliferation<br>Extensive proliferation
Proliferation of blood vessels	0: Normal<br>1: Slight<br>2: Moderate<br>3: Severe	 Mild proliferation<br>Moderate proliferation<br>Extensive proliferation

Supplementary Figures

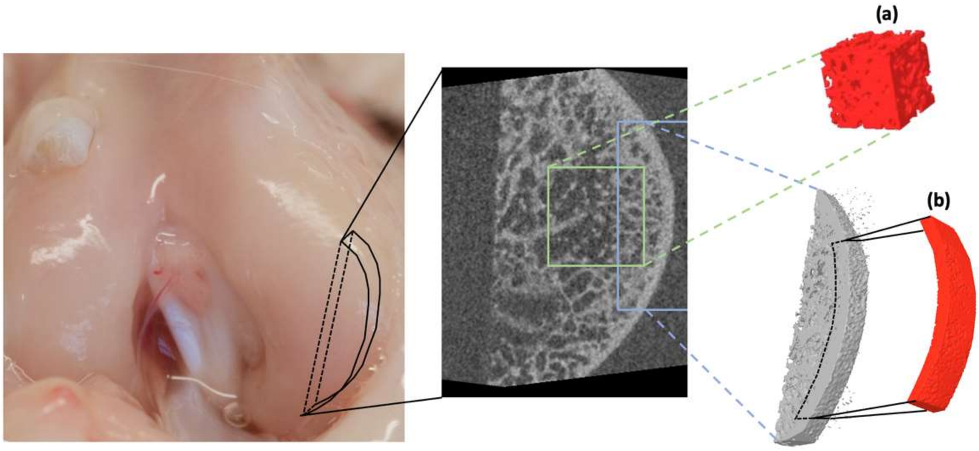

Figure S1: Assessment of the condylar bone by µCT. To assess BV/TV, Tb.Th, Tb.Sp, and Tb.N, an area of 2 mm^3 of the trabecular bone was defined in the medial condyle (see (a)). For the assessment of cBM and cTh, a defined area with a width of 1.9 mm, a height of 11 mm, and a depth of 1 mm was chosen (see (b)).

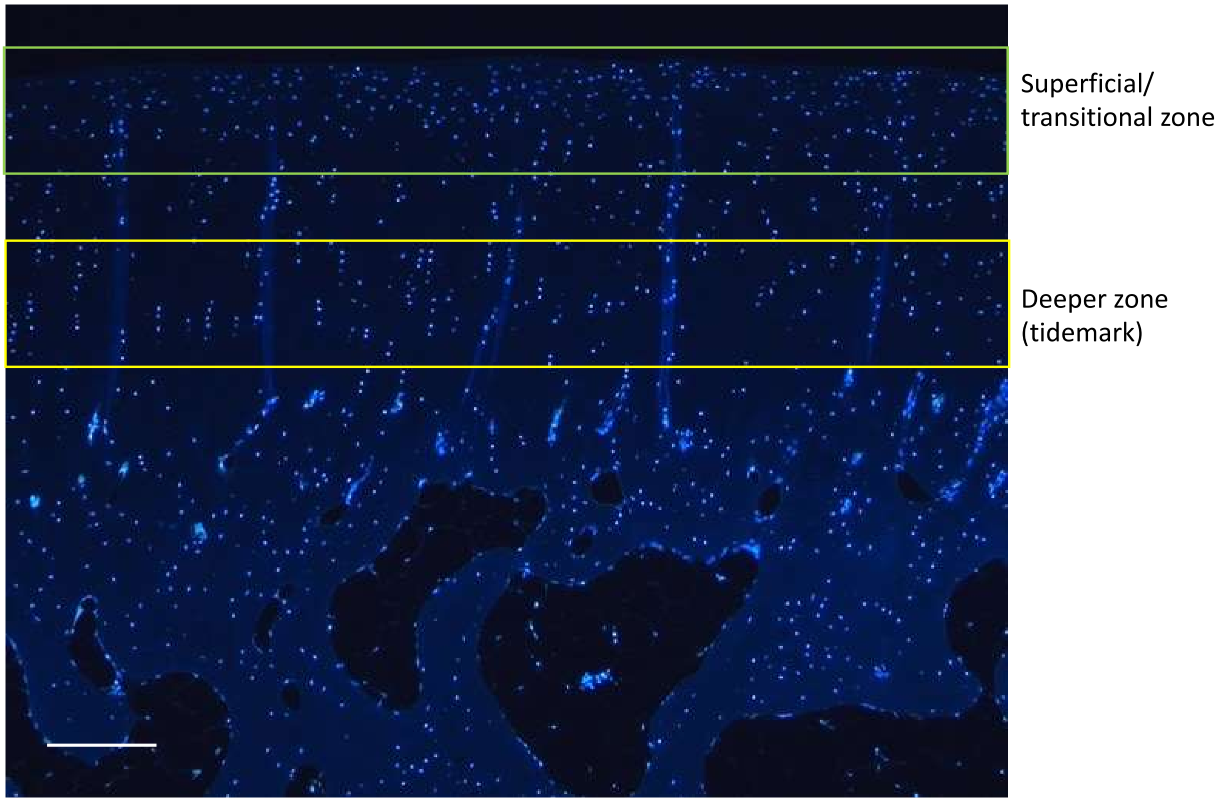

Figure S2: Zones considered in the TUNEL assay. Images were taken from the superficial/ transitional zone (green box) as well as from the deeper zone/ tidemark region (yellow box). The white bar represents 200 µm.

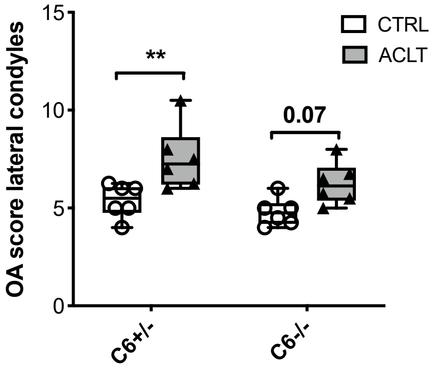

Figure S3: Histopathologic assessment of lateral femoral condyle sections. Statistical analysis of the overall score, comprising proteoglycan content/ SafO staining intensities, structure/ surface integrity, hypocellularity, and cluster formation. Data are charted as box plots with median and whiskers min to max; white boxes= control joint, grey boxes= ACLT-operated joints; n=6.

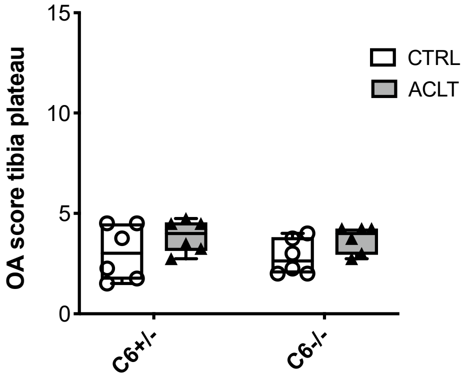

Figure S4: Histopathologic assessment of tibia plateau (medial + lateral) sections. Statistical analysis of the overall score, comprising proteoglycan content/ SafO staining intensities, structure/ surface integrity, hypocellularity, and cluster formation. Data are charted as box plots with median and whiskers min to max; white boxes= control joint, grey boxes= ACLT-operated joints; n=6.

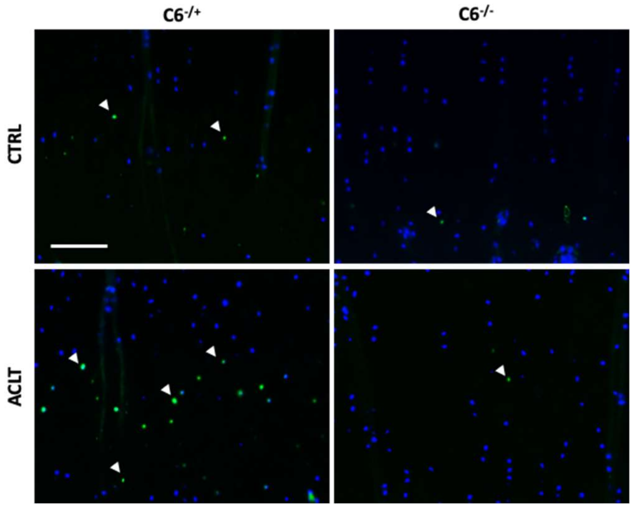

Figure S5: TUNEL staining. Exemplary images of TUNEL staining taken from the deep zone (tidemark). White arrowhead= TUNEL-positive cell (green); the white bar represents 100 µm; n= 3.
